# A Novel Alkaline Phosphatase/Phosphodiesterase, CamPhoD, from Marine Bacterium *Cobetia amphilecti* KMM 296

**DOI:** 10.3390/md17120657

**Published:** 2019-11-22

**Authors:** Yulia Noskova, Galina Likhatskaya, Natalia Terentieva, Oksana Son, Liudmila Tekutyeva, Larissa Balabanova

**Affiliations:** 1 Laboratories of Marine Biochemistry and Bioassays and Mechanisms of Action of Biologically Active Substances, G.B. Elyakov Pacific Institute of Bioorganic Chemistry, Far Eastern Branch, the Russian Academy of Sciences, Vladivostok 690022, Russia; galin56@mail.ru (G.L.);; 2 School of Economics and Management of Far East Federal University, Vladivostok 690950, Russia

**Keywords:** recombinant alkaline phosphatase, bimetal-dependent phosphodiesterase, marine bacterium, *Cobetia amphilecti*, PhoD

## Abstract

A novel extracellular alkaline phosphatase/phosphodiesterase from the structural protein family PhoD that encoded by the genome sequence of the marine bacterium *Cobetia amphilecti* KMM 296 (CamPhoD) has been expressed in *Escherichia coli* cells. The calculated molecular weight, the number of amino acids, and the isoelectric point (pI) of the mature protein’s subunit are equal to 54832.98 Da, 492, and 5.08, respectively. The salt-tolerant, bimetal-dependent enzyme CamPhoD has a molecular weight of approximately 110 kDa in its native state. CamPhoD is activated by Co^2+^, Mg^2+^, Ca^2+^, or Fe^3+^ at a concentration of 2 mM and exhibits maximum activity in the presence of both Co^2+^ and Fe^3+^ ions in the incubation medium at pH 9.2. The exogenous ions, such as Zn^2+^, Cu^2+^, and Mn^2+^, as well as chelating agents EDTA and EGTA, do not have an appreciable effect on the CamPhoD activity. The temperature optimum for the CamPhoD activity is 45 °C. The enzyme catalyzes the cleavage of phosphate mono- and diester bonds in nucleotides, releasing inorganic phosphorus from *p*-nitrophenyl phosphate (pNPP) and guanosine 5′-triphosphate (GTP), as determined by the Chen method, with rate approximately 150- and 250-fold higher than those of bis-pNPP and 5′-pNP-TMP, respectively. The Michaelis–Menten constant (K_m_), V_max_, and efficiency (k_cat_/K_m_) of CamPhoD were 4.2 mM, 0.203 mM/min, and 7988.6 S^−1^/mM; and 6.71 mM, 0.023 mM/min, and 1133.0 S^−1^/mM for pNPP and bis-pNPP as the chromogenic substrates, respectively. Among the 3D structures currently available, in this study we found only the low identical structure of the *Bacillus subtilis* enzyme as a homologous template for modeling CamPhoD, with a new architecture of the phosphatase active site containing Fe^3+^ and two Ca^2+^ ions. It is evident that the marine bacterial phosphatase/phosphidiesterase CamPhoD is a new structural member of the PhoD family.

## 1. Introduction

Alkaline phosphatases are widely distributed in marine bacteria, which release inorganic phosphate (P_i_) from phosphorus-containing compounds dissolved in the ocean and utilize them for their own growth and reproduction [1,2,3]. Globally, marine bacteria and diatoms have been shown to store and concentrate P_i_ and then release it into the local marine environment using phosphatase (Pho) activity, thus biologically inducing and controlling phosphorite and apatite nucleation [4,5]. Moreover, the enzymes from marine sources have flexible molecular structures, and therefore work better at ambient or lower temperatures, which opens the possibility to decrease temperatures of production processes for their application in biotechnology [6,7,8,9].

Currently, three large families of prokaryotic alkaline phosphatases are known, namely PhoA, PhoD, and PhoX. They differ from each other in structure, substrate specificity, and the dependence on different metal ions for the manifestation of their activities [10]. It has been shown that the PhoD-like phosphatases belonging to the phosphatase/phosphodiesterase family more commonly occur in marine and soil bacteria than PhoA and PhoX [11]. This is due to the presence in the environment of available sources of phosphorus and cofactors in their habitat. It has been proven that the PhoD family is common in the bacteria living in phosphorus- and metal-depleted conditions [3,5,11]. Previously, natural and recombinant alkaline phosphatases isolated from marine bacteria have been described, but information about their alkaline phosphodiesterases is still lacking [6,12,13,14,15].

There are many families of phosphodiesterases, which include phospholipases C and D, autotaxin, sphingomyelin phosphodiesterase, DNases, RNases, and restriction endonucleases. However, phosphodiesterases usually refer to the cyclic nucleotide phosphodiesterases degrading cyclic adenosine and guanosine monophosphates (cAMP and cGMP). According to the primary structure and differences in the catalytic domains, they are divided into three known classes: (1) the eukaryotic enzymes; (2) enzymes such as phosphodiesterases from the yeast *Dictyostelium* and the bacteria *Vibrio*; and (3) the bacterial enzymes homologous to purple acid phosphatases and dimetallophosphoesterases, including the three subclasses A, B, and C [16]. The bacterial phosphodiesterases were isolated and characterized from *Aphanothece halophytica* [9], *Delftia acidovorans* [17], *Sphingobium* sp. TCM1 [18], *E. coli* [19], *B. subtilis* [20], as well as a novel unclassified enzyme from the metagenome of an Indian coalbed [16]. All these metal-dependent phosphodiesterases showed maximal activities in the alkaline pH range, and needed different metal ions, such as Ca^2+^, Zn^2+^, Mg^2+^, or Mn^2+^, for their catalytic activity. The isolated phosphodiesterases were capable of cleaving phosphoric acid residues from specific substrates, such as Bis-*p*-nitrophenyl phosphate (Bis-pNPP) and thymidine-5′-monophosphate-*p*-nitrophenylester (5′-pNP-TMP), which are mostly used as DNA models for studies of phosphodiester hydrolysis [16,21]. It has been previously assumed that the role of PhoD-like enzymes is to participate in the nucleic acid exchange in cells during the main metabolism, taking into account their ability to hydrolyze the phosphodiester bonds [22].

Among three types of phosphoester bonds existing in nature (mono-, di-, and triester), the phosphodiester bond is exceptionally stable, with a half-life of approximately 3 × 10^7^ years at a moderate temperature and a neutral pH, while an acceleration of its cleavage up to 10^16^-fold in biological processes can be achieved through enzymatic hydrolysis of this bond by the highly specialized metalloenzymes, such as nucleases and phosphoesterases [23,24]. However, finding and exploring novel enzymes with phosphoesterase activity is still a challenge in biotechnology because of some their inherent limitations, such as undesirable selectivity, difficulties in extraction or synthesis, high cost, narrow functional temperature, and pH range [25]. The phosphoesterase function in nature may be related to hydrolyzing a wide range of biomolecules (proteins, nucleic acids, and lipids) implicated in DNA repair, post-translational modification, biomineralization, and energy metabolism, as well as in signal transduction through regulation of the circulation of secondary metabolites, particularly free nucleotides and their analogues [26,27]. The phosphodiesterase families are mostly considered to have a common catalytic domain pocket, with the universal mechanism of nucleophilic attack to control the intracellular levels of cyclic nucleotides, and to be regulators of many physiological and pathophysiological processes [28]. Due to their important role in intracellular signal transduction and the possibility of finding their exact subcellular localization, phosphodiesterases are considered very attractive pharmacological targets [29,30]. Therefore, there is a growing interest in finding ways to disrupt, block, or manipulate quorum sensing (QS) signaling in bacteria [29]. The producers of QS signals have been found among both the free-living and associated marine bacteria inhabiting invertebrates and algae [31]. Consequently, they are promising sources for new bioactive compounds, such as the QS modulators or inhibitors [29].

The QS-related phosphodiesterases of marine origin have yet to be investigated. However, two alkaline phosphatases of the juvenile *Euprymna scolopes* light organ were found to play an active role in dephosphorylating lipid A of the luminous marine bacterium *Vibrio fischeri,* which changes its signaling properties in relation to the host tissues during their symbiotic colonization [32]. The PhoA alkaline phosphatase (CmAP) of the marine bacterium *C. amphilecti* KMM 296 (Collection of Marine Microorganisms, G.B. Elyakov Pacific Institute of Bioorganic Chemistry, Far Eastern Branch, the Russian Academy of Sciences (PIBOC FEB RAS)) isolated from the coelomic fluid of the mussel, *Crenomytilus grayanus,* was suggested to promote the host mollusk shell’s mineralization and biofilm regulation of many species of food-derived pathogens [6,7,12]. The mechanism of the CmAP biological action is still unclear and remains under investigation.

Thus, it has been recently shown that the biological role of alkaline phosphatases is more complex and broader than previously assumed. Alkaline phosphatases appear to be involved in major cellular events, such as the regulation of protein phosphorylation, cell growth, apoptosis, and cellular migration [32,33,34]. Therefore, most human conditions or diseases are accompanied by a change in the level of alkaline phosphatase expression, which is the basis of diagnostics. A newly discovered function of alkaline phosphatases is in maintaining tissue and organ homeostasis by inactivation of bacterial lipopolysaccharides (LPS), and by regulation of cell secretion, microbiome and tumor behavior, and possibly detoxication of hyperphosphorylated extracellular tau proteins, which play a key role in progression of Alzheimer’s disease [32,33,34]. Recently, bovine and human intestinal recombinant alkaline phosphatases underwent clinical trials related to inactivating LPS and preventing inflammation for the treatment of surgical diseases and metabolic disorders [33,34,35]. It is possible that the search for marine enzymes with dephosphorylating activity and the study of their mechanism of action will also present an application for the new treatment.

The genome sequence analysis of *C. amphilecti* KMM 296 has shown that the bacterium produces not only the highly active alkaline phosphatase CmAP, belonging to the PhoA family (GenBank ID: WP_084589490.1), but also the functionally active PhoD-like phosphatase/phosphodiesterase (GenBank ID: WP_043333989.1), with a novel structure and properties [6,12,36]. This article presents the results of isolation of the gene encoding for the PhoD-like protein from *C. amphilecti* KMM 296, and in producing the recombinant enzyme CamPhoD with the alkaline phosphatase and phosphodiesterase activities and properties.

## 2. Results and Discussion

### 2.1. CamPhoD Isolation and Characterization by Enzymatic Activity and Primary Structure

**Figure 1:**
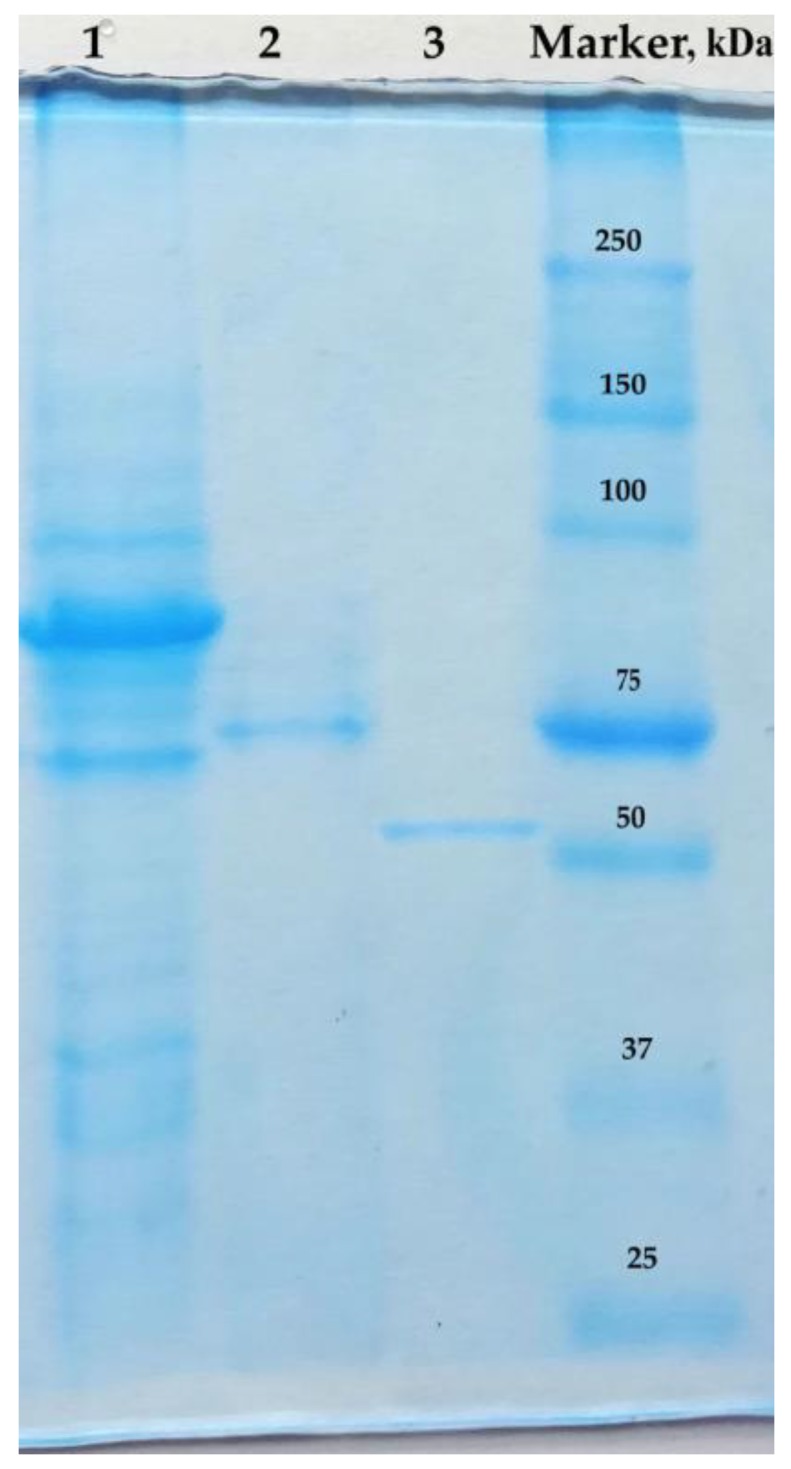
SDS-PAGE image of CamPhoD. Line 1-the crude extract from the recombinant *E. coli* cells; line 2-the purified CamPhoD before the enterokinase treatment; line 3-the purified CamPhoD after the treatment with enterokinase to remove the 34.2 kDa His-tagged N-end of the plasmid pET 40b(+) sequence (shaperon DsbC); line 4-the marker of the protein molecular weights (BioRad).

The heterologous expression of the *C. amphilecti* KMM 296 gene (GenBank ID: WP_043333989) corresponding to the open reading frame (ORF) of the PhoD-like phosphatase (CamPhoD) resulted in obtaining an enzymatically active recombinant protein with a specific phosphatase activity of 18.2 U/mg (with p-NPP as a substrate) after purification using the modified scheme described earlier [6]. The CamPhoD phosphodiesterase activity at the cleavage of bis-pNPP was 0.3 U/mg. The isolation of this enzyme confirmed the ability of *C. amphilecti* to produce the functionally active alkaline bifunctional phosphatase/phosphodiesterase CamPhoD with a calculated molecular weight of 54839.8 kDa for the mature protein, without the 33-letter leader peptide, according to the Simple Modular Architecture Research Tool (SMART) database [37]. The obtained data were in agreement with the polyacrylamide gel electrophoresis (PAGE) estimation of its molecular weight (Figure 1).

Apart from the leader peptide indicating an extracellular intent of the enzyme, the CamPhoD 492 amino acid (aa) sequence includes the region of the fibronectin type III repeat (FN3) from 43 to 118 aa residues, containing a cell recognition region of Arg-Gly-Asp (RGD) in a flexible loop between 2 strands, according to the new functional classification of proteins via subfamily domain architectures [38]. RGD is the cell attachment site of a large number of adhesive extracellular matrix and cell surface proteins, which are recognized by transmembrane receptors activating signal transduction. FN3-like domains were also found in bacterial glycosyl hydrolases [38]. It has been shown that the bacterial extracellular proteins with the RGD motif may be located on the surface of the bacterial type IV secretion pili. They mimic fibronectin in triggering cell spreading, focal adhesion formation, and activation of several tyrosine kinases during interaction with various mammalian cell lines [39,40]. Thus, the RGD motif of CamPhoD may be a player in pathogenesis or the symbiotic relationships between the bacterium and host mollusk during its shell mineralization [12,36]. The part of the CamPhoD molecule from 149 to 505 aa residues is the PhoD-like phosphatase (pfam09423), with characteristic active and ion-binding sites [38].

The values of CamPhoD-specific activities corresponded to the activities of the PhoD phosphatase/phosphodiesterase from *Aphanothece halophytica* and alkaline phosphatase from *Vibrio* sp., whose molecular weights were also similar to CamPhoD and other alkaline phosphatases [10,41,42,43,44,45]. Thus, the molecular weights of the alkaline phosphatases’ monomers from *Streptomyces griseus* IMRU 3570, *Pyrococcus abyssi*, and *Thermotoga maritima* were 62 kDa, 54 kDa, and 45 kDa, respectively [42,43,44]. The enzymes with exclusively phosphodiesterase-related activity possess subunits with lower molecular weight, such as in the enzyme ZiPD from *E. coli* (36 kDa) [45]. Alkaline phosphatases generally have a dimeric structure, but the literature also describes a trimer for the phosphodiesterase from *Delftia acidovorans* with a molecular weight of 85 kDa [17]. According to the gel chromatography data, the native CamPhoD tended to form an active dimer with a molecular weight of approximately 100–120 kDa in the conditions used (see Experimental Procedure section).

### 2.2. Expression Conditions for CamPhoD Production

**Figure 2:**
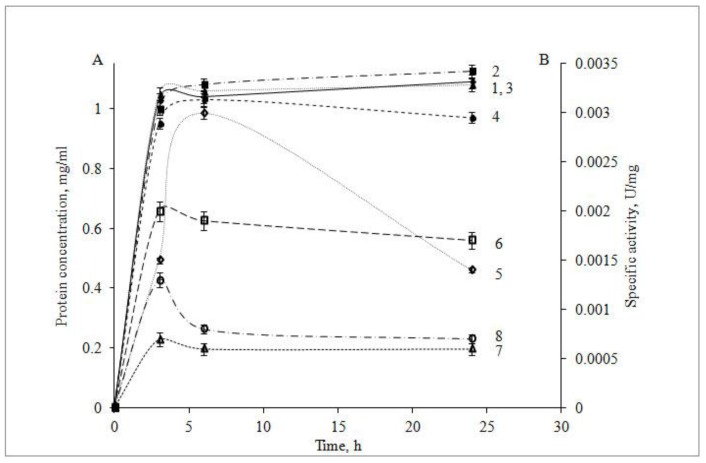
Determination of the expression conditions for the recombinant CamPhoD production: (**A**) the dependence of the CamPhoD concentration (mg/mL) on the cultivation time at 37 °C in the presence of (1) 0.1 mM isopropyl β-D-1-thiogalactopyranoside (IPTG), (2) 0.2 mM IPTG, (3) 0.3 mM IPTG, and (4) 0.5 mM IPTG; (**B**) the dependence of the CamPhoD phosphatase activity (U/mg) on the cultivation time at 37 °C in the presence of (1) 0.1 mM IPTG, (2) 0.2 mM IPTG, (3) 0.3 mM IPTG, and (4) 0.5 mM IPTG.

The study of optimal expression conditions for the recombinant CamPhoD showed that its highest yield was achieved when cultivating the recombinant *E. coli* strain over 6 h at 37 °C, with an addition of 0.1 mM isopropyl β-D-1-thiogalactopyranoside (IPTG) in the Luria–Bertani (LB) medium (Figure 2). It was also found that the alkaline phosphatase activity of the recombinant CamPhoD increased when the cells were cultured in a medium depleted by phosphorus. The growth of the recombinant *E. coli* cells in a MX medium containing 80 mM KH_2_PO_4_ described earlier [46] significantly reduced the alkaline phosphatase activity of CamPhoD down to 0.035 U/mg compared with the activity of the enzyme, which was produced in the standard phosphate-free LB medium, similarly to the phoD phosphatase isolated from *Streptomyces coelicolor* [47].

### 2.3. Physicochemical and Enzymatic Properties of CamPhoD

**Figure 3:**
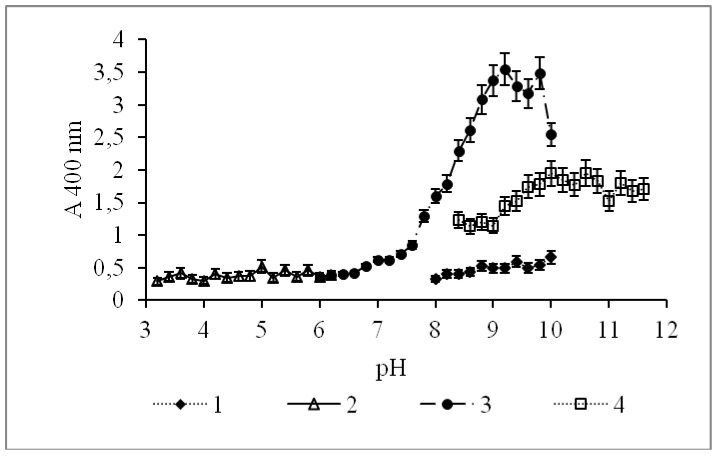
Effect of the pH value on the CamPhoD phosphatase activity for conducting the enzymatic reaction in various buffer solutions: (1) glycine buffer, (2) acetate buffer, (3) Tris-HCl buffer, and (4) bicarbonate buffer.

The optimum pH for the CamPhoD maximum activity was determined to be 9.2, which lies in the range of pH 8.0–11.0 inherent for most alkaline phosphatases and phosphodiesterases (Figure 3). For comparison, the phosphodiesterases from *Sphingobium* sp. TCM1 and *E. coli* exhibited the maximal activity at pH 9.5 and 8.5–9.8, respectively [18,19].

**Figure 4:**
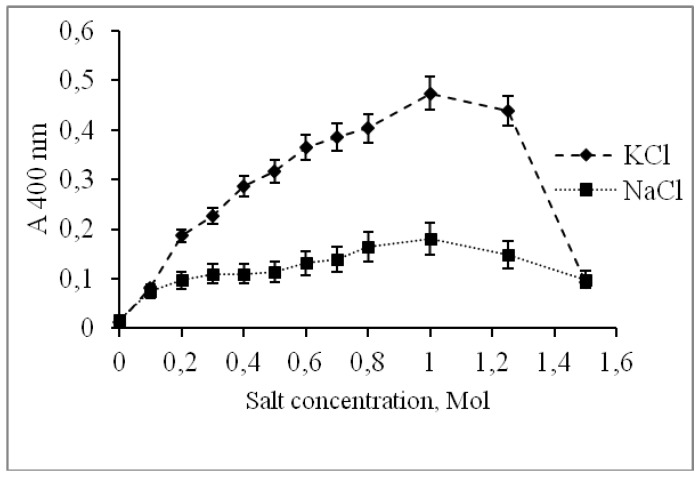
Effect of NaCl and KCl on the CamPhoD alkaline phosphatase activity.

The significant effect of KCl on the activity and the absence of activity without any salt in the incubation medium indicated that CamPhoD was a highly salt-tolerant enzyme, which is a common trait of many enzymes of marine origin [6,9,48,49]. Moreover, the PhoD alkaline phosphatase/phosphodiesterase from a halotolerant cyanobacterium, *Aphanothece halophytica*, has been shown to be induced and secreted out of cells by salt stress [9]. In the presence of KCl in a concentration up to 1 M, CamPhoD was 2–3 times more active than in the presence of NaCl at the same concentration (Figure 4).

Concentration of both KCl and NaCl of more than 1 M decreased the CamPhoD activity. A similar effect was noted for the salt-tolerant alkaline phosphatase from eggs of the sea urchin *Strongylocentrotus intermedius* [48,50] and the enzyme from the halophilic bacterium *Halomonas* sp. 593 [49].

It was found that the addition of Co^2+^ and Mg^2+^ to the incubation mixture containing the lysate with the recombinant CamPhoD after ultrasonic homogenization of the *E. coli* cells increased its activity by 100% compared to the control. The addition of CaCl_2_ and FeCl_3_ activated CamPhoD from the cell lysate by 70% and 60%, respectively. For the CamPhoD purified up to level of homogenous protein, the salts of divalent metals Zn ^2+^, Mn ^2+^, and Cu ^2+^ at a concentration of 2 mM had almost no effect on its ability to cleave pNPP, while the salts of Co ^2+^, Mg ^2+^, Ca ^2+^, Fe ^3+^, and Ni ^2+^ drastically activated the enzyme, indicating its metal dependence (Table 1).

However, the maximal CamPhoD activity was achieved by the simultaneous addition of Co^2+^ and Fe^3+^ to the incubation mixture, whereas only an 80% or 50% increase of the activity was observed after the addition of Co^2+^ and Fe^3+^ separately. It is evident that CamPhoD is the first bimetal Co^2+^–Fe^3+^-dependent phosphatase/phosphodiesterase characterized to date. Previously, the PhoD-like phosphatase from *B. subtilis* was described as being closely related to purple acid phosphatases (PAPs) with tyrosinate-ligated Fe^3+^ ions, but differed from them by having two Ca^2+^ ions instead of a single extra Fe^2+^, Mn^2+^, or Zn^2+^ ion [15]. The Ca^2+^ dependence of the phosphatase/phosphodiesterase from *Aphanothece halophytica* [9] as well as the activation of the alkaline phosphatase from *Pyrococcus abyssi* in the presence of Mg^2+^, Zn^2+^, and Co^2+^ ions have already been shown [43]. In addition, the alkaline phosphatase of the hyperthermophilic bacterium *Termatoga maritima* was shown to contain Co^2+^ and Mg^2+^ in the active center [44], while the active center of an *E. coli* alkaline phosphatase possessing the phosphodiesterase activity contained two Zn^2+^ and one Mg^2+^ [51], similar to most of the PhoA alkaline phosphatases, for example CmAP from *C. amphilecti* KMM 296 [6,24,52].

In spite of the obvious CamPhoD metal-dependence, the addition of EDTA and EGTA to the incubation medium at a concentration of 2 mM led to almost no effect on its activity, probably due to the deeply hidden metal-binding site in the core domain, a common trait of extracellular marine enzymes [6]. The narrow enzymatic cavity directed towards the catalytic aa residues, which is packed with metal ions, protects the extracellular *C. amphilecti* KMM 296 alkaline phosphatase CmAP from the damaging effects of chelating agents [6].

**Figure 5:**
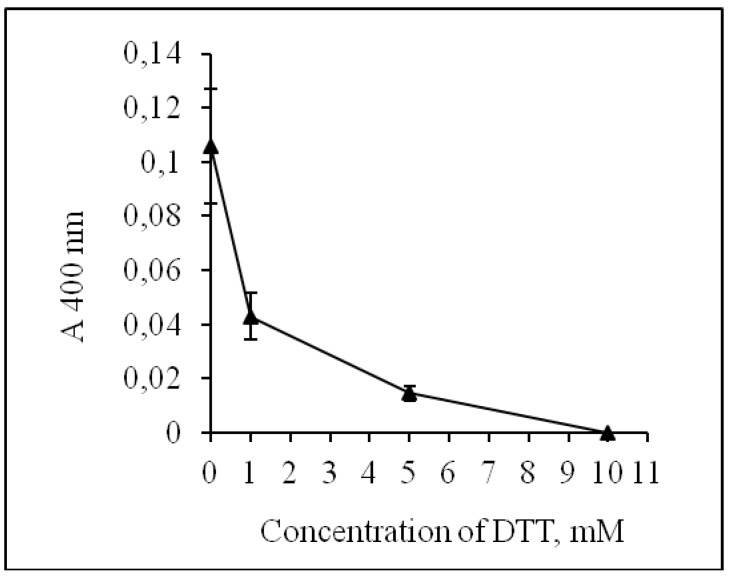
Effect of dithiothreitol (DTT) on the CamPhoD phosphatase activity.

The treatment of CamPhoD with dithiothreitol (DTT) at a concentration of 10 mM completely inhibited enzyme activity (Figure 5). The sensitivity to sulfhydryl reagent has been shown previously for the highly active alkaline phosphatase CmAP, which was previously isolated from the same bacterium *C. amphilecti* KMM 296 [6]. This indicates that the presence of SH groups in the protein structure is necessary for enzyme activity, although CmAP does not have any intermolecular disulfide bond [6,12].

Adding the non-ionic detergent Triton X-100 to the CamPhoD incubation mixture at concentrations of 1%, 0.1%, and 0.01% also reduced its activity by 80%, 70%, and 46%, respectively. This could influence the hydrophobic interactions in the protein, the importance of which were shown for the overall thermal stability of psychrophilic and mesophilic enzymes [53].

**Figure 6:**
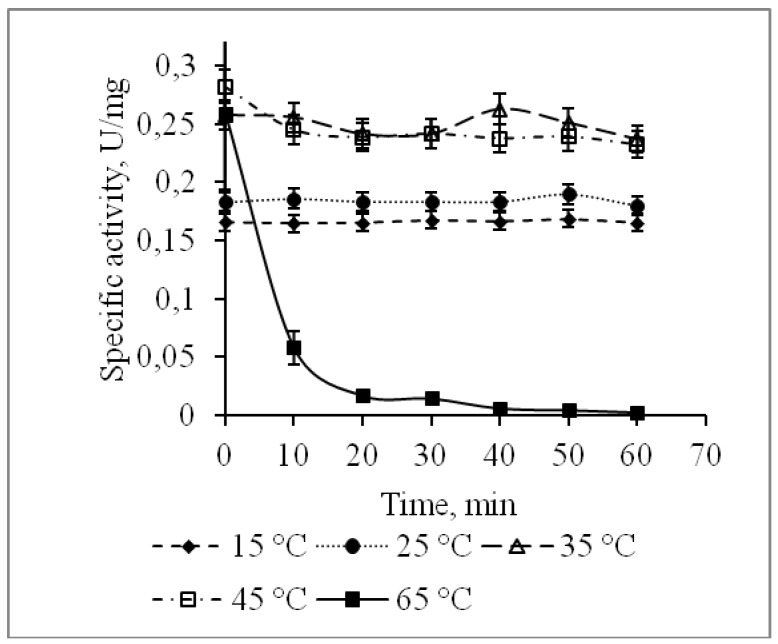
Effect of temperature on the CamPhoD stability.

**Figure 7:**
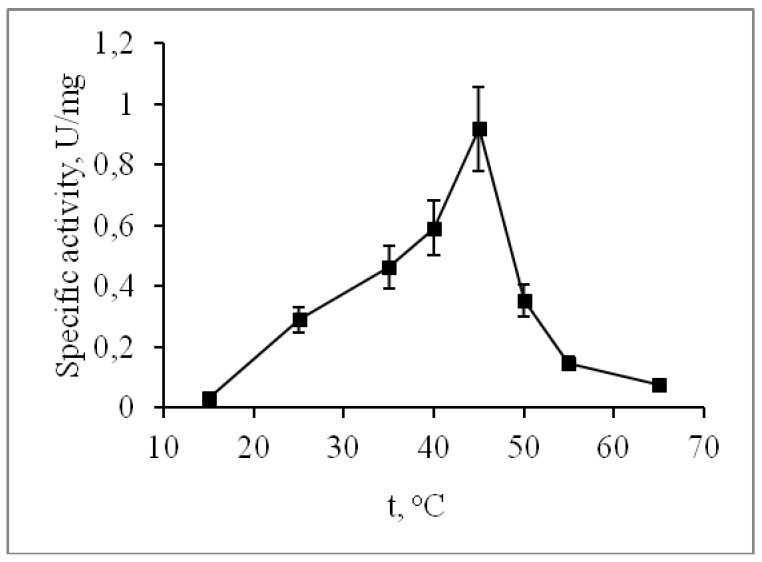
Effect of temperature on the CamPhoD phosphatase activity.

The alkaline phosphatase CamPhoD retained its activity during incubation for 60 min at temperatures ranging from 15 to 45 °C, while incubation at 65 °C completely inhibited its activity after 20 min (Figure 6). The CamPhoD thermostability and the optimal temperature of 45 °C (Figure 7) are similar to the CmAP properties [6]. As for phosphatases and phosphodiesterases, their temperature optimums cover a wide range, allowing them to belong to both thermolabile and thermostable enzymes. Alkaline phosphodiesterases from *Sphingobium* sp. and *Delftia acidovorans* exhibit maximum activity at 55 °C and 65 °C, respectively [17,18].

### 2.4. Substrate Specificity of CamPhoD

The study on substrate specificity of CamPhoD has shown that the enzyme catalyzes the cleavage of the phosphate group from deoxy- and ribonucleoside mono-, -di-, and-triphosphates in the following order according to catalytic rate: pNPP ≥ CMP ≥ GTP ≥ UMP ≥ dCMP ≥ AMP ≥ TMP ≥ CTP ≥ GDP ≥ GMP ≥ UDP = CDP ≥ bis-pNPP ≥ 5′-pNP-TMP (Table 2). According to the Chen method [54] of free phosphorus determination, P_i_ was released from pNPP and GTP under the CamPhoD catalysis approximately 150 and 250 times faster than from bis-pNPP and 5′-pNP-TMP, respectively (Table 2). The CamPhoD activities toward the chromogenic substrates pNPP, bis-pNPP, and 5′-pNP-TMP obtained by the spectrophotometric determination of the p-nitrophenyl concentration were similar. The ability of CamPhoD to cleave the phosphate group of the diesters bis-pNPP and 5′-pNP-TMP, as well as different phosphate monoesters, allowed us to assign this enzyme to the bifunctional phosphatase/phosphodiesterase [9,10,16,17,18,19,20,55]. For comparison, the binuclear zinc phosphodiesterase ZiPD from *E. coli* possessed activity towards phosphodiester bonds of bis-pNPP and 5′-pNP-TMP only, and did not catalyze the cleavage of other phosphates, such as AMP, ADP, ATP, cyclic phosphates, or nucleic acids [45]. Despite a wide variety of nucleoside mono-, di-, and triphosphates used as substrates, CamPhoD did not catalyze the cleavage of the λ DNA, plasmid pUC19 DNA (Thermo Scientific, Vilnus, Lithuania), oligonucleotides (Evrogen, Moscow), or c-di-GMP (Sigma) up to inorganic phosphorus Pi (Table 2), similar to the phosphodiesterase PhoD from *B. subtilis* [15].

### 2.5. Catalytic Properties of CamPhoD

The Michaelis constant (K_m_) of the alkaline phosphatase/phosphodiesterase CamPhoD at pH 9.0 in the presence of Co^2+^ and Fe^3 +^ with the use of chromogenic pNPP as a substrate was 4.2 mM, the maximum velocity (V_max_) was 0.203 mM/min, and the efficiency (k_cat_/K_m_) was 7988.6 S^−1^/mM. Using the chromogenic phosphate bis-pNPP, Km was determined to have values of 6.71 mM, V_max_ = 0.046 mM/min, and efficiency (k_cat_/K_m_) = 1133.0 S^−1^/mM. The kinetic parameters obtained for CamPhoD are similar to those previously obtained for other phosphatases and phosphodiesterases (Table 3). For example, K_m_ of the alkaline phosphatase from *Termatoga maritima* had a value of 175 mM [44], while K_m_ of the alkaline phosphodiesterases from *Sphingobium* sp. TCM1 and *A. halophytica* for pNPP were 1.5 mM and 3.38 mM, respectively [10,18].

The CamPhoD catalytic efficiency (*k*_cat_/*K*_m_) in relation to pNPP was seven-fold higher than that with the use of bis-pNPP as the substrate, indicating that the enzyme mainly has a phosphomonoesterase structure with phosphodiesterase capability, similar to other PhoD-like enzymes [15]. However, the value of its catalytic efficiency for diester bonds is much higher when compared with many monospecific phosphodiesterases, excluding the enzyme from an unknown protein family with recently established structure and properties, which was isolated from the metagenome [18,19,45].

### 2.6. 3D Modeling of CamPhoD

**Figure 8:**
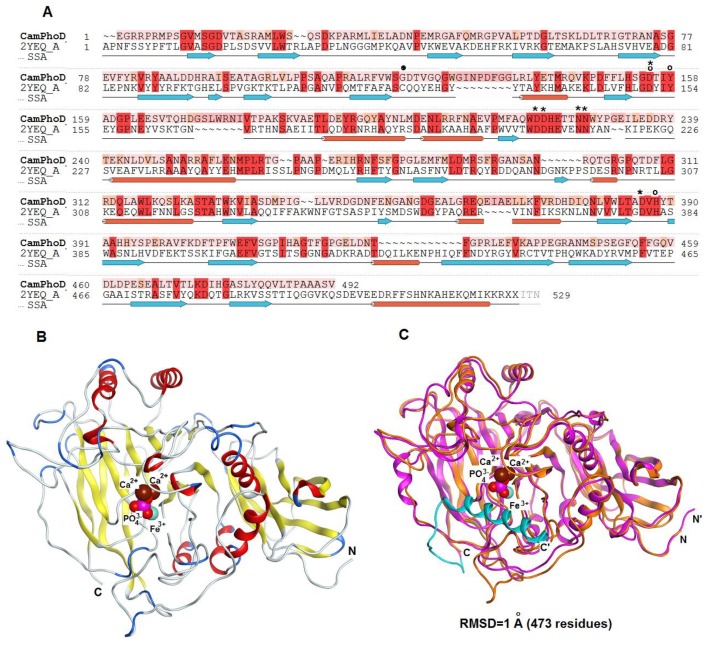
(**A**) Modeling of the CamPhoD 3D structure. An alignment of the amino acid sequences of the alkaline phosphatase/phosphodiesterase CamPhoD from the marine bacterium *C. amphilecti* KMM 296 (GenBank ID: WP_043333989) and alkaline phosphatase (phosphodiesterase) D from *B. subtilis* (PDB ID: 2YEQ). The amino acid sequences identity and similarity (color boxed) and the secondary structure of the template are highlighted. Note: α-helixes = red sticks; β-structure = blue arrows; the binding of amino acid (aa) residues (Ca2+/Co2+) = *; the binding of conserved aa residues (Fe^3+^) = o; and the residue Cys 124 of the template = •. (**B**) The 3D structure model of CamPhoD with the reaction product Pi and metal ions in the active center (the protein structure is a ribbon diagram, Pi is in stick form, and Ca^2+^ is shown as spheres). (**C**) The 3D superimposition of the CamPhoD model (orange) and the template (PDB ID: 2YEQ) (shown in pink, with the blue C-terminal part).

A theoretical model for the PhoD-like phosphodiesterase/phosphatase from the marine bacterium *C. amphilecti* KMM 296 (GenBank ID: WP_043333989) was generated using structural bioinformatics methods (Figure 8A–C). Among the 3D structures currently available in the protein database (PDB), the low identical alkaline phosphatase D from *B. subtilis* (PDB ID: 2YEQ), which has a new architecture of the phosphatase active site based on Fe^3+^ and two Ca^2+^ ions, is apparently a single homologous template for modeling CamPhoD, which is a new member of the PhoD family inherent in the marine bacteria (data not shown). The amino acid sequences of CamPhoD and the template possess 20.5% identity and 38% similarity. (Figure 8A). The aa residues of the CamPhoD binding Ca^2+^/Co^2+^ atoms in the active center are highly conserved. However, the bonds of the iron atom in the CamPhoD active center differ from the template bonds in replacing Cys 124 with Gly 117 (Figure 8A). In comparison with the template, the phosphate molecule in the modeled CamPhoD active site interacts with Tyr 158, Asp 221, and with one Fe^3+^ atom, two Ca^2+^ atoms, and three water molecules (Figure 8C).

**Figure 9:**
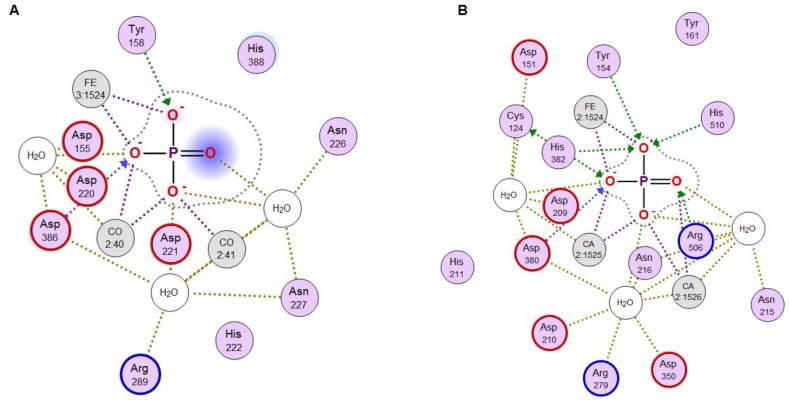
A 2D diagram of the contacts of the CamPhoD active center (**A**) and the template (PDB ID: 2YEQ) (**B**).

A similar interaction with phosphate is observed when two Ca^2+^ atoms are replaced with Co^2+^ (Figure 9). The superimposition of the CamPhoD model and template showed that the root mean square deviation (RMSD) for 473 Cα atoms is 1 Å. The structural differences are in the structure of some of the loops on the outer surface of the protein (Figure 8C). In the CamPhoD structure, there is no α-helix at the C-terminus of the molecule, which is presented in the template, which possibly regulates the availability of the active center to accept the substrate (Figure 8A,C).

**Figure 10:**
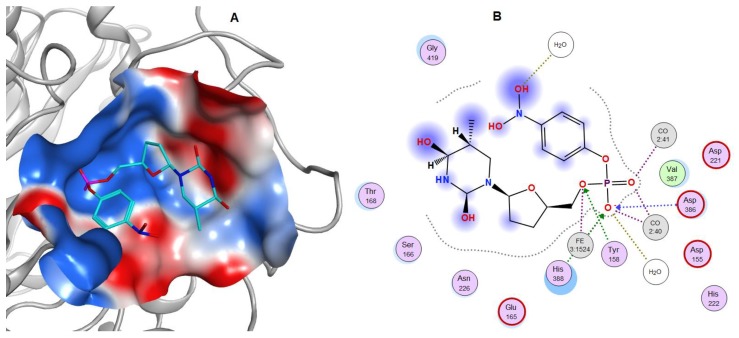
A model of the CamPhoD complex with the substrate 5′-pNP-TMP (**A**)**,** and a 2D diagram of the contacts of 5′-pNP-TMP in the CamPhoD active center (**B**).

The analysis of contacts in the CamPhoD complexes with the reaction product PO_4_^3−^ showed that the enzyme forms fewer contacts with the product than the template due to the shortened C-terminal region (Figure 9). The model of the CamPhoD complex with the substrate molecule has made it possible to determine the amino acid residues of the enzyme associated with the substrate binding (Figure 10).

### 2.7. Effect of CamPhoD on Bacterial Biofilms

In order to study the effect of alkaline phosphatase/phosphodiesterase CamPhoD on the inhibition of biofilm formation or on their destruction, bacterial biofilms of both individual and mixed species were grown. The study found that CamPhoD (0.1 U/mg) had a slight inhibitory effect on the biofilm formation of three species of *Bacillus*, namely *B. licheniformis*, *B. aegricola*, and *B. berkelogi* (18–32%), and dispersed the already formed biofilms of these species by 8–15% (Table 4). At the same time, CamPhoD did not inhibit the formation of biofilms in *B. subtilis* and *Pseudomonas aeruginosa* and did not degrade them.

Under natural conditions, biofilms are most often formed by not just one but by several types of bacteria [56]. In view of this, the study of such mixed biofilms is of great fundamental and practical importance. We investigated the formation of biofilms by mono and mixed cultures of *Yersinia pseudotuberculosis* and *Salmonella enteritidis*, as well as the effect of CamPhoD during the 3 day incubation with the enzyme (Table 4). The destruction of mature biofilms by the studied enzyme ranged from 11% to 24% depending on the strain. For comparison, DNase I degraded about 30% of the biofilm formed by *Y. pseudotuberculosis* and more than half of the *B. subtilis* biofilm [57].

The alkaline phosphatase CmAP from *C. amphilecti* KMM 296 was also shown to effectively inhibit the growth of the new biofilms and degradation of the mature biofilms of *S. enteritidis,* as well as *P. aeruginosa* and *B. subtilis* [7], in contrast to CamPhoD. The antibacterial activity of the alkaline phosphatase against *P. aeruginosa* has been found in *E. coli*, which is known as a causative agent of diarrhea [58]. The effect of alkaline phosphatases on pathogens has been studied by exploring the gut microbiota modulation ability in the alkaline phosphatase of the intestinal PhoA enzymes, in which the level of decrease or dysfunction is associated with intestinal inflammation, dysbiosis, bacterial translocation, and subsequently systemic inflammation [33,34,35].

The presence of extracellular alkaline phosphatases of the structural families PhoA (GenBank ID: WP_084589490.1) and PhoD (GenBank ID: WP_043333989.1) in the marine gamma-proteobacterium *C. amphilecti* KMM 296 may indicate either their distinct or cooperative functions for the hydrolysis of various phosphorus-containing organic molecules, depending on the environmental conditions and cell lifestyle [7,36]. The analogous PhoD enzyme from *B. subtilis* is thought to target specific phosphate-containing molecules, such as teichoic acids linked to the wall peptidoglycan via phosphodiester bonds. It is possible that other PhoD family members also have specific biological roles rather than operating as general phosphatases, such as members of the PhoA and PhoX families [15]. In spite of the absence of both CamPhoD and CmAP ability to cleave an important signaling molecule c-di-GMP, they may provide a level of Pi, acting on other extracellular phosphate-containing substrates and playing a crucial role in the bacterial behavior. For example, the phosphorylated lipid A or the linear intermediate product of the c-di-GMP hydrolysis, pGpG, have also been recently found as substrates for alkaline phosphatases and phosphodiesterases and as participants in cell signaling [32,59]. The mechanism of the putative participation of any alkaline phosphatase from *C. amphilecti* KMM 296 in the bacterial cell signaling has yet to be clarified, including regulation of biofilms or the species content in the bacterial consortium, with which the marine bacterium has to coexist in the mollusk digestive system.

## 3. Materials and Methods

### 3.1. Reagents and Materials

The reagents of chemical grade qualifications were obtained from Merck (Munchen, Germany), Sigma (Sigma-Aldrich Rus LLC, Moscow, Russia), and Helicon (Moscow, Russia). The molecular biology kits for restriction, ligation, Taq polymerase, and oligonucleotides were from Evrogen (Moscow, Russia) and Thermo Fisher Scientific RU (Moscow, Khimki, Russia); kanamycin was from Sintez (Moscow, Russia). Yeast extract, bactoagar, tripton, and pepton were from Helicon and Dia-M (Moscow, Russia). DNA and protein molecular weight markers were from BioRad (California, USA).

### 3.2. Construction of Plasmid pET40CamPhoD

The recombinant plasmid pET40CamPhoD was constructed by insertion into the NcoI/SacI fragment of plasmid pET-40b (+) (Thermo Fisher Scientific-RU, Moscow, Khimki, Russia), the gene encoding for the PhoD-like full-length phosphatase/phosphodiesterase, which was synthesized by polymerase chain reaction (PCR) using the genomic DNA of *Cobetia amphilecti* KMM 296 (Collection of Marine Microorganisms PIBOC FEB RAS) as a template and the gene-specific primers CamPhoD-NcoI-dir: 5′ -TATACCATGGAAGGACGGCGCCCGCGCATGCCCTC-3′ and CamPhoD-SacI-rev: 5′ -TATAGAGCTCTTAGACACTGGCGGCGGCGGGGGTC-3′.

The reaction conditions were: 1 µL 10 × Encyclo buffer, 0.2 µl 50 × Encyclo polymerase mixture (Encyclo PCR kit; Evrogen, Moscow, Russia), 0.2 µl 50 × dNTP mixture (10 mM of each), a mixture of primers (1 µl 5 µM of each), and 1 µl 20 ng DNA. The volume of the reaction mixture was 10 μL. The amplification process consisted of 40 cycles of PCR (15 s, 95 °C, 1 min 40 s, 72 °C). After amplification, the PCR product was purified by electrophoresis in 1% agarose gel. The PCR fragment (1 μg) was treated with the restriction enzymes NcoI and SacI in an optimal buffer (Thermo Fisher Scientific RU) for 3 h at 37 °C, and then the enzymes were removed from the reaction mixture using phenol (1: 1). Here, 1/10 volume of 0.3 M sodium acetate, pH 5.2, and 1/2 volume of isopropyl alcohol were added to the aqueous fraction containing the PCR fragment, then incubated at −20 °C for 30 min. Then, this was centrifuged at 14,000 rpm for 20 min, the precipitate was washed with 75% ethanol, then dried at room temperature. The precipitate was dissolved in 20 μl of deionized water.

In total, 2 μg of the pET-40b (+) plasmid DNA (Thermo Fisher Scientific-RU) was treated with the NcoI and SacI restriction endonucleases in accordance with the procedure described above.

The obtained fragment of the CamPhoD gene and the NcoI /SacI part of the plasmid pET-40b (+) were ligated using a ligase reaction in 50 μl of ligation buffer, according to the instructions (Thermo Fisher Scientific RU). Then, 10 μL of the reaction mixture was used to transform the competent *E. coli* Rosetta cells (DE3). Transformants were grown on the Luria–Bertani (LB) agar containing 25 µg/mL kanamycin. After incubation for 16 h at 37 °C, the clones were screened, and then the targeted plasmid DNA was isolated and screened for mutations.

### 3.3. Optimization of Conditions for CamPhoD Expression

To determine the optimal IPTG concentration, the *E. coli* Rosetta (DE3) cells transformed with the pET40 plasmid carrying the CamPhoD gene were grown on LB agar containing 25 mg/mL kanamycin overnight at 37 °C. Single colonies were selected and grown in 5 mL of the liquid LB medium containing 25 mg/mL kanamycin at 200 rpm for 16 h at 37 °C. Then, the inoculum was placed into the flasks with 20 mL of fresh LB medium containing kanamycin at a concentration of 25 mg/mL and incubated at 37 °C in a shaker at 200 rpm, up to an optical density 0.6–0.8 (λ 600 nm). Next, 0.1 mM, 0.2 mM, 0.3 mM, and 0.5 mM IPTG were added to induce the expression of CamPhoD, then incubation was continued at 37 °C. To determine the phosphatase activity, 5 mL of each sample was taken at 0, 3, 6, and 24 h after the start of expression and ultra-sonication for the bacterial cells and determination of the CamPhoD alkaline phosphatase activity.

To determine the dependence of CamPhoD activity on the presence of phosphate in the growth medium, the *E. coli* Rosetta (DE3) cells transformed with the pET40CamPhoD plasmid were grown in 25 mL of the liquid medium containing: bacto-trypton 10 g/L, yeast extract 7.5 g/L, sorbitol 70 g/L, MgCl_2_ 5 mM, KH_2_PO_4_ 80 mM, and 25 mg/mL kanamycin at 200 rpm for 16 h at 37 °C [46]. Then, the cells were placed in 1 L of the fresh medium of the abovementioned composition and incubated at 37 °C on a rocking chair at 200 rpm, up to an optical density 0.6–0.8 (λ 600 nm). Next, 0.1 mM IPTG was added to induce the expression of CamPhoD and incubation was continued at 37 °C for 6 h at 200 rpm. After this, CamPhoD was isolated, purified, and its activity was determined as described below.

### 3.4. The Recombinant CamPhoD Production

The recombinant *E. coli* Rosetta (DE3) strain was grown in 25 mL of the LB liquid medium containing 25 mg/mL kanamycin at 200 rpm for 16 h at 37 °C. Then, the cells were placed in the fresh LB medium (1 L) containing kanamycin at a concentration of 25 mg/mL and incubated at 37 °C in a shaker at 200 rpm, up to an optical density at 600 nm of 0.6–0.8. After that, 0.1 mM IPTG was added to induce expression of the enzyme and incubation was continued at 37 °C for 6 h at 200 rpm.

The cells were precipitated by centrifugation at 4000 rpm for 15 min at 8 °C, suspended in 35 mL of 25 mM Tris-HCl buffer (pH 9.0) with phenylmethylsulfonyl fluoride (PMSF) added to a final concentration of 0.15 mM, and subjected to ultrasonication at 22 kHz and 0–4 °C with intervals of 30 s, up to clarification of the suspension. The suspension was centrifuged at 11,000 rpm for 30 min at 8 °C, the precipitate was discarded, and the activity and properties of CamPhoD were determined in the resulting extract.

### 3.5. The Recombinant CamPhoD Isolation and Purification

For isolation of CamPhoD, CaCl_2_ and MgCl_2_ were added to the recombinant cell extract to a final concentration of 10 mM, DNase was (SkyGen, Moscow, Russia) to a final concentration of 5 μg/mL, these were incubated at 37 °C for 1 h, and then centrifuged at 11000 rpm for 20 min. The resulting supernatant was introduced into a 25 × 3.2 cm Ni-IMAC-Sepharose column (GE Healthcare Life Sciences, Buckinghamshire, UK) equilibrated with 25 mM Tris-HCl, pH 9.0 (buffer A), and washed with five volumes of the same buffer. The recombinant protein was eluted with a linear gradient of 0–0.5 M imidazole in 25 mM Tris-HCl buffer, pH 9.0, and 0.5 M NaCl (6 column volume), at a rate of 1.3 mL/min. The CamPhoD-containing fraction was purified on a 10 × 1.4 cm Source 15 Q column (GE Healthcare Life Sciences) equilibrated with buffer A, then the protein was eluted with a linear gradient of 0–0.5 M NaCl in the 25 mM Tris-HCl buffer at pH 9.0. Ion exchange chromatography was performed at a rate of 1 mL min; the volume of the fractions was 1 mL. The CamPhoD-containing fractions were collected and treated with enterokinase at a final concentration of 1 U per 1 mg of protein for 22 h at 25 °C. Then, the protein solution was applied to a Superdex 200 PG column (105 × 2 cm) (GE Healthcare Life Sciences), previously equilibrated with buffer A with 0.15 M NaCl at a rate of 0.5 mL/min, with 1 mL fractions. The CamPhoD-containing fractions were collected and subjected to chromatography using a mono-Q HR column (4 × 0,8 cm) (GE Healthcare Life Sciences, Buckinghamshire, UK) equilibrated with buffer A, washed with 10 volumes of buffer A, and then the target protein was eluted with a linear gradient of 0–0.5 M NaCl in buffer A at a rate of 0.5 mL/min and with fractions of 1 mL. The purified preparation of CamPhoD was used to study the physicochemical properties and substrate specificity.

### 3.6. Enzyme Activity Assay

The enzyme activity of CamPhoD was determined at 37 °C for 30 min with the use of 2 mM p-nitrophenyl phosphate (*p*-NPP), thymidine-5′-monophosphate-p-nitrophenylester (5′-pNP-TMP) or bis-p-nitrophenyl phosphate (Bis-*p*-NPP) as chromogenic substrates in 25 mM Tris-HCl buffer, pH 9.0, 2 mM CoCl_2_, 2 mM FeCl_3_. The volume of the reaction mixture was 0.5 mL. The reaction was stopped by adding 1 mL of cooled 0.5 M NaOH to the reaction mixture. The absorption of the formed p-nitrophenol was measured at an optical density of 400 nm. The amount of enzyme required for the conversion of 1 μM *p*-nitrophenyl from a substrate over 1 min was taken as a unit of activity. The specific activity is given in units calculated per 1 mg of protein. The protein concentration was determined using Bradford’s method [60].

### 3.7. Substrate Specificity

The substrate specificity of CamPhoD was determined by adding 2 mM CMP, CDP, CTP, dCMP, dCTP, UMP, UDP, UTP, TMP, TTP, GMP, GDP, GTP, dGMP, dGTP, AMP, ATP, dAMP, bis-pNPP, 5′-pNP-TMP, c-di-GMP, and pNPP to a standard incubation mixture, containing 25 mM Tris-HCl buffer, pH 9.0, 2 mM CoCl_2_, 2 mM FeCl_3_. The mixture was incubated at 37 °C for 60 min. The volume of the reaction mixture was 1 mL. Then, a molybdate reagent with ascorbic acid (4 mL) was added to the incubation mixture and again incubated at 37 °C for 60 min. The mixture was cooled to room temperature, and absorbance of the formed product of dephosphorylation by CamPhoD was measured at an optical density of 820 nm. The amount of inorganic phosphate P_i_ (mkM) released during dephosphorylation of the studied substrates with the enzyme was determined using a calibration curve with KH_2_PO_4_, according to Chen’s method [54].

### 3.8. Determination of Thermostability and Temperature Optimum

The effect of temperature on CamPhoD and the optimum temperature were determined by incubating the standard incubation mixture at temperatures ranging from 15 to 65 °C, at 10 °C intervals. Alkaline phosphatase activity was determined using the standard method for determining for CamPhoD, as described above.

### 3.9. Effect of Metal Ions and Chelating Agents

The influence of divalent metal ions on CamPhoD was determined using the standard method for determining the alkaline phosphatase activity, with the addition of Mg^2+^, Ca^2+^, Mn^2+^, Zn^2+^, Co^2+^, Ni^2+^, Cu^2+^, Cs^2+^, Fe^3+^ ions, EDTA, and EGTA at a concentration of 2 mM to the incubation mixture. The incubation mixture without cations and chelating agents was used as a control.

### 3.10. Effect of NaCl and KCl

The effects of Na^+^ and K^+^ were investigated by adding NaCl and KCl to a standard incubation mixture at a concentration of 0–1.5 M. The alkaline phosphatase activity was determined as described above.

### 3.11. Determination of Molecular Weight

The molecular weight of CamPhoD was determined using gel filtration on a calibrated Superdex G200 PG (GE Healthcare Life Sciences, Amersham Place, Little Chalfont, Buckinghamshire, HP7, 9NA, UK) column (105 × 2 cm) and polyacrylamide gel electrophoresis under denaturing conditions (sodium dodecyl sulfate polyacrylamide gel electrophoresis) using the Lammley method [61].

### 3.12. Determination of Catalytic Parameters

Kinetic parameters were calculated by plotting the rate of p-NPP and bis-p-NPP splitting at concentrations from 5 to 15 mM in a buffer containing 25 mM Tris-HCl, pH 9.0, 2 mM CoCl_2_, 2 mM FeCl_3_. The reaction was carried out at 25 °C. The Michaelis constant K_m_, the maximum reaction rate V_max_, and the turnover number k_cat_ were determined by plotting the Layuver–Burk graph using the OriginPro 8.5 program.

### 3.13. Molecular Modeling

The target template alignment customization of the modeling process and 3D model building for CamPhoD (GenBank: WP_043333989) were carried out using the Molecular Operating Environment (MOE) version 2018.01 package, using the Amber12: EHT forcefield (EHT-Extended Hueckel Theory) [62]. The alkaline phosphatase D from *B. subtilis* (PDB code: 2YEQ) was used as a template, which had a high-resolution crystal structure. The evaluation of structural parameters, contact structure analysis, physicochemical properties, and visualization of the results were carried out with the ligand interaction and dock modules in the MOE 2018.01 program [62].

### 3.14. Biofilms Growth and Enzymatic Treatment

The strains of Bacillus subtilis, Bacillus licheniformis, Bacillus aegricola, Bacillus berkelogi, Pseudomonas aeruginosa, Yersinia pseudotuberculosis, and Salmonella enteritidis were used. An overnight bacterial culture was diluted with the appropriate nutrient medium and incubated in a 96-well plate at 200 μl per well for 24 h at 37 °C, or for 3 days at 22–24 °C. After incubation, loose cells were removed from the wells, and wells were washed three times with 0.85% NaCl. The biofilm was stained with 0.5% crystal violet (CV) for 20 min at room temperature. The dye was removed from the wells, and unbound CV was washed with tap water. The plates were air dried, 2% acetic acid in 95% ethanol was added to each well, and the absorbance was determined at 600 nm.

Inhibition of biofilm formation was tested using the method in [63]. Test substances at various concentrations were added to the wells. All experiments were repeated four times. The destruction of biofilms by the investigated substances was carried out as follows. After the formation of biofilms for a certain time, unattached cells were removed and the wells were washed with 0.85% NaCl. The enzyme was added to each well in the appropriate buffer and incubated under the conditions for determination of the activity of the studied enzyme. Then, the plate was processed as described above.

## 4. Conclusions

The PhoD-like enzyme gene was firstly isolated from the marine bacterium *Cobetia amphilecti* KMM 296 and cloned into *E.coli*. The effects of chemicals, metal ions, kinetic parameters, and substrate specificity on the enzymatically active recombinant product, CamPhoD, expressed from this gene confirmed that the enzyme carries a metabolic function of phosphatase/phosphodiesterase of the PhoD family in the marine environment. The enzyme bimetal dependence coincides with the modeling results for the enzymatic complex, with phosphate in the active center surrounded by two Co^2+^ ions and one Fe^3+^ ion. This enzyme of marine origin has been concluded to be a new member of the bifunctional PhoD-like phosphatase/phosphodiesterase class, with a characteristic structure and important biological functions.

## Figures and Tables

**Table 1:** Effect of metal cations on the alkaline phosphatase CamPhoD from *Cobetia amphilecti* KMM 296.

Metal Cations, 2 mM	Retained Activity, (%) *
Co ^2+^	80
Mg ^2+^	68
Ca ^2+^	50
Fe ^3+^	48
Ni ^2+^	32
Cs ^2+^	23
Mn ^2+^	20
Zn ^2+^	18
Cu ^2+^	7
Co ^2+^ + Fe ^3+^	100
Ca ^2+^ + Fe ^3+^	66

* The various metal ions were added to the reaction mixture at the 2 mM salt concentration. The phosphatase activity was measured using the standard method described in Experimental Procedures. The retained activity is expressed as the specific phosphatase activity of CamPhoD relative to the activity of the enzyme incubated in the control reaction mixture in the absence of any metal ion.

**Table 2:** Substrate specificity of the alkaline phosphatase/phosphodiesterase CamPhoD from *Cobetia amphilecti* KMM 296 *.

Substrate	Amount of Released P_i_, mkM
pNPP	0.52
GTP	0.475
CMP	0.465
UMP	0.37
dCMP	0.325
AMP	0.31
TMP	0.305
CTP	0.24
GDP	0.19
GMP	0.16
UDP	0.15
CDP	0.14
dGMP	0.12
UTP	0.12
dCTP	0.09
dAMP	0.055
TTP	0.05
dGTP	0.04
c-di-GMP	0.005
bis-pNPP	0.003
5′-pNP-TMP	0.002
*λ* DNA	0
pUC19	0
Oligonucleotides	0

* These results were obtained using a molybdate reagent with ascorbic acid [54]. Various substrates were added to the reaction mixture at 2–15 mM concentrations. For each substrate, a control consisting of a specific substrate and a buffer containing 25 mM Tris-HCl (pH 9.0), 2 mM CoCl_2_, and 2 mM FeCl_3_ was used.

**Table 3:** Biochemical characteristics of the reported bacterial phosphatases and phosphodiesterases.

Strain (Enzyme)	Optimum		bis-pNPP			p-NPP			Ref.
	t (°C)	pH	K_m_ (mM)	*k*_cat_ (S^−1^)	k_cat_/K_m_ (S^−1^/mM)	K_m_ (mM)	*k*_cat_ (S^−1^)	k_cat_/K_m_ (S^−1^/mM)	
***Cobetia amphilecti* KMM 296 (CamPhoD)**	45	9.2	6.7	7603.2	1133.0	4.2	33552	7988.6	
***C. amphilecti* KMM 296 (CmAP)**	40- 50	10.3	-	-	-	13.2	28300	2144	[6]
***Aphanothece halophytica***	-	10	3.13	-	-	3.38	-	-	[10]
***Bacillus subtilis***	25	8.0	-	-	-	0.05	1.2	24	[15]
***Metagenome***	25	8.5	10.21	615 × 10^4^	602 × 10^3^	-	-	-	[16]
***Delftia acidovorans***	65	10	2.9	52740	18186.2	5.0	10260	2052	[17]
***Sphingobium* sp. TCM1**	55	9.5	6.1	325	53.3	1.5	37.9	25.3	[18]
***Escherichia coli* (YfcE)**	-	9.8	9.74	19.8	2.03	-	-	-	[19]
***Termatoga maritima***	75	8.0				175	16	0.091	[44]
***E. coli* (*ElaC*, ZiPD)**	-	-	4	59	14.75	-	-	-	[45]

**Table 4:** Effect of the alkaline phosphatase/phosphodiesterase CamPhoD from *Cobetia amphilecti* KMM 296 on the bacterial biofilms.

Strain	Biofilm Formation % (3 Days, 22–24 °C)	Biofilm Destruction % (30 min, 37 °C)
K *	100	0
*B. subtilis*	100	0
*B. licheniformis*	74	15
*B. aegricola*	68	14
*B. berkelogi*	82	8
*P. aeruginosa*	100	0
*Y. pseudotuberculosis*	85	23
*S. enteritidis*	93	11
*Y. pseudotuberculosis + S. enteritidis*	87	24

* K-control strains grown without treatment with the enzyme.
